# Clinical significance of aberrant mammalian target of rapamycin expression in stage IIIB colon cancer

**DOI:** 10.3892/ol.2014.2285

**Published:** 2014-06-25

**Authors:** MEILING WEN, BAOXIU LI, XIAOFEI CAO, CHENGYIN WENG, YONG WU, XISHENG FANG, XIAOSHI ZHANG, GUOLONG LIU

**Affiliations:** 1 Department of Medical Oncology, Affiliated Nanhua Hospital, Nan Hua University of South China, Hengyang, Hunan 421002, P.R. China; 2 Department of Oncology, Guangzhou First People’s Hospital, Guangzhou Medical University, Guangzhou, Guangdong 510180, P.R. China; 3 State Key Laboratory of Oncology in South China, Cancer Center, Sun Yat-Sen University, Guangzhou, Guangdong 510275, P.R. China

**Keywords:** mammalian target of rapamycin, colon cancer, prognosis, molecular target therapy

## Abstract

The aim of the present study was to investigate the significance of aberrant expression of mammalian target of rapamycin (mTOR) and the activated form of mTOR kinase, phosphorylated mTOR (pmTOR), in human stage IIIB colon cancer. The expression of mTOR and pmTOR was detected by immunohistochemistry in the tumor tissue of stage IIIB colon cancer patients. The association between the expression of mTOR, pmTOR and clinicopathological parameters of patients was analyzed. The positive expression of mTOR and pmTOR was observed to be higher in 75.5% (80/106) and 76.4% (81/106) of the 106 colon cancer specimens, compared with the adjacent normal tissues. The high level of pmTOR expression was found to be significantly higher in the invasive tumor front cells and resulted in a higher risk of mortality. The results suggested that mTOR and pmTOR may be promising clinical markers and present novel molecular targets for designing novel therapeutic strategies to treat this malignancy.

## Introduction

Colorectal cancer is the third most common type of malignant tumor worldwide (1). The diagnosis of patients in the early stages of the disease has been found to exhibit a positive effect with regard to improving overall survival. However, the outcomes of patients diagnosed with advanced-stage disease remains relatively poor despite the use of novel chemotherapy drugs (2–4). Thus, there is a critical requirement to improve the understanding of molecular biomarkers associated with the advanced stage of colorectal carcinomas to devise treatment strategies and improve clinical outcomes.

Mammalian target of rapamycin (mTOR) is a Ser/Thr protein kinase, which has been recognized as a central regulator of cell proliferation and angiogenesis (5). mTOR kinase is important in a number of pathways involved in cancer and metabolic diseases (6). In several solid tumors, activation of the mTOR pathway has been found to correlate with the promotion of cell proliferation, differentiation, apoptosis evasion and resistance to cytotoxic therapy in cancer cells (7–10). In the present study, the expression of mTOR and phosphorylated mTOR (pmTOR, the active form of mTOR) was examined in stage IIIB colon cancer, and their correlation with clinicopathological characteristics was investigated, to provide prognostic value and potential molecular targets for future novel therapies.

## Materials and methods

### Patients and specimens

Tumor and corresponding adjacent normal tissues were obtained from 106 patients with stage IIIB (T3-4N1M0) colon carcinoma, who underwent curative surgery without prior treatment at Guangzhou First People’s Hospital (Guangzhou, China) between January 2001 and July 2007. The study was approved by the ethics committee of Guangzhou First People’s Hospital and patients provided written informed consent. Tissues were fixed in formalin, paraffin-embedded and diagnosed clinically and histopathologically. The study included 69 males and 37 females, whereby 58 individuals were ≥60 years old and 48 individuals were <60 years old (median age, 54 years). A total of 64 cases of carcinoma were identified on the left-side of the colon and 42 cases on the right-side of the colon (divided by the splenic flexure of colon boundaries). Of these patients, 83 cases exhibited papillary adenocarcinoma and tubular adenocarcinoma, and 23 cases exhibited mucinous adenocarcinoma and signet-ring cell carcinoma. Furthermore, 21 cases of poorly differentiated carcinoma were identified, as well as 80 cases of moderately differentiated carcinoma and five cases of well-differentiated carcinoma. In addition, 93 cases were staged as T_3_N_1_M_0_ and 13 cases were staged as T_4_N_1_M_0,_ according to the 6th American Joint Committee on Cancer staging system (11). Patients were followed up for at least five years according to National Comprehensive Cancer Network guidelines (12). Follow-up evaluations were conducted via telephone and letter, to obtain information on the patients’ outcomes. The follow-up deadline was February 2013. The survival time was calculated from the final date of disease diagnosis to the follow-up deadline or date of mortality, which was predominantly due to carcinoma recurrence. The median follow-up duration was 55 months (range, 9–102 months).

### Immunohistochemistry

The resected stage IIIB colon specimens were fixed in 4% formaldehyde and cut into 4-μm slices. Slides were deparaffinized in xylene twice for 10 min and rehydrated using descending concentrations of ethanol. Antigen retrieval was performed in 0.01 mol/l citrate buffer (pH 6.0) using a microwave oven for 10 min at 98–100°C. Endogenous peroxidase activity was blocked using 3% hydrogen peroxide (in fresh methanol) for 10 min at room temperature. Following washing with phosphate-buffered saline, the sections were incubated with blocking serum for 1 h. Next, the tissue sections were stained for primary polyclonal rabbit anti-human antibodies against mTOR (Abcam, Cambridge, UK) and pmTOR (Cell Signaling Technology, Inc., Danvers, MA, USA), respectively. Specimens were then incubated with the primary antibody overnight at 4°C. Horseradish peroxidase-conjugated goat anti-rabbit polyclonal IgG (Zymed, Beijing, China) was used as the secondary antibody. Positive staining was visualized using 3,3′-diaminobenzidene.

### Evaluation of score

mTOR and pmTOR protein expression was evaluated by two pathologists without knowledge of the clinical patient data. Images were captured using an Olympus BX41 light microscope (Olympus Corporation, Tokoyo, Japan). Tumor cells exhibiting cytoplasmic and/or membrane immunohistochemical expression were considered positive cells. The percentage of positive tumor cells was counted in five separate fields and ≥1,000 adjacent cells in the area with the highest density of positive cells for each slide. The extent of staining was scored as follows: 0, <5%; 1, 5–25%; 2, 26–50%; 3, 51–75%; and 4, >75% of cells in the respective lesions. The intensity of staining was scored as follows: 0, negative (no brown staining); 1, weak (light brown staining); 2, moderate (intermediate brown staining); and 3, strong (dark brown staining). The two values obtained were multiplied to calculate a receptor score (maximum value, 12). Scores between 6 and 12 were defined as preserved or strong staining pattern (+++), scores between 4 and 6 were defined as middle staining pattern (++), scores of 2 or 3 were defined as weak staining pattern (+) and scores of 0 or 1 were defined as negative expression (−). For statistical analysis, the samples were grouped into positive (score, >3) or negative (score, ≤3) groups.

### Statistical analysis

All statistical analyses were performed using SPSS statistical software, version 13.0. (SPSS, Inc., Chicago, IL, USA). The non-parametric Kendall’s Tau-b test was applied to analyze the correlations between clinicopathological parameters. Five-year overall survival curves were obtained using the Kaplan-Meier method and compared using the log-rank test. The Cox proportional-hazards model was used for multivariate analysis. All tests were two-tailed and P<0.05 was considered to indicate a statistically significant difference.

## Results

### mTOR and pmTOR expression in stage IIIB colon cancer

**Figure 1:**
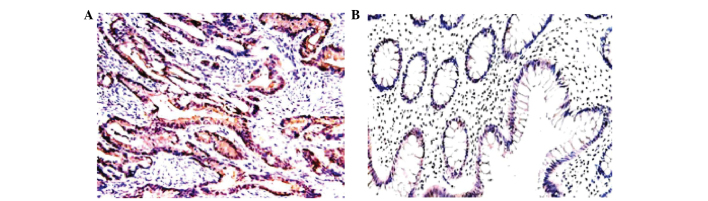
Expression of mTOR in stage IIIB colon cancer. The expression of mTOR in (A) tumor tissue and (B) adjacent normal mucosa was examined using immunohistochemistry (magnification, ×200). mTOR, mammalian target of rapamycin.

**Figure 2:**
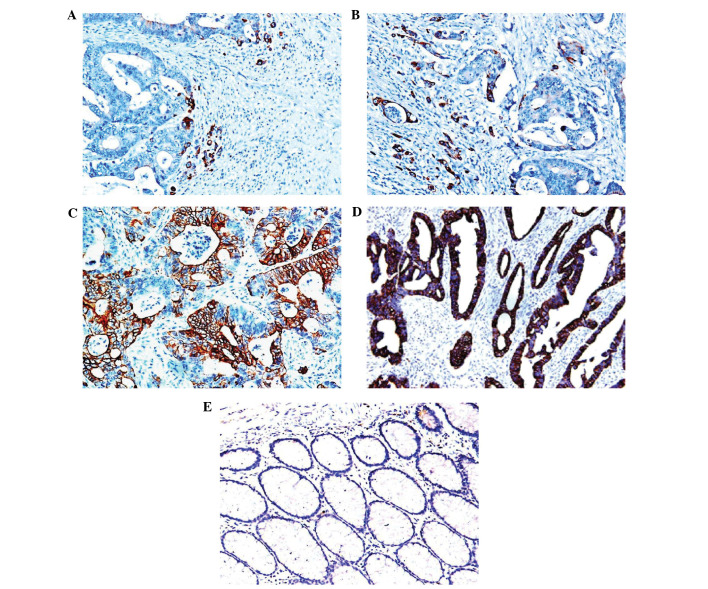
Expression of pmTOR in stage IIIB colon cancer. The expression of pmTOR in (A–D) tumor tissue and (E) adjacent normal mucosa was examined using immunohistochemistry (magnification, ×200). The expression of pmTOR (A and B) at the invasive tumor front cell and (C and D) in the central/superficial section of colon cancer cells. pmTOR, phosphorylated mammalian target of rapamycin.

The expression of mTOR and pmTOR was analyzed by immunohistochemistry in 106 clinical specimens of stage IIIB colon cancer. mTOR and pmTOR were found to be expressed in the cell membrane and cytoplasm of tumor tissues (Fig. 1A and B). Out of the total 106 specimens, the positive rates of mTOR and pmTOR expression were 75.5% (80/106) and 76.4% (81/106), respectively. By contrast, mTOR and pmTOR were not expressed in the normal mucosa samples obtained from colon cancer patients (Fig. 2A and B). A total of 76 cases were found to highly express both mTOR and pmTOR, and four and five cases were found to highly express only mTOR or pmTOR, respectively. A high expression of pmTOR at the tumor borders was observed in 28 cases, whereas 39 cases exhibited a high expression of pmTOR in the center of tumors, and 14 cases exhibited high pmTOR expression at the tumor border and tumor center.

### Correlation between mTOR and pmTOR expression and clinicopathological characteristics

Using the non-parametric Kendall’s Tau-b statistical test, a positive correlation was identified between the expression of mTOR and pmTOR (r=0.786; P<0.001). Furthermore, pathological type was found to significantly correlate with pathologic classification (r=0.275; P=0.010). However, partial correlational analysis identified no significant correlation between mTOR and pmTOR expression and clinicopathological features, including age, gender, tumor location, pathological type and TNM stage (Table I).

### Univariate and multivariate analysis of five-year overall survival of colon cancer patients

Survival analysis was performed for all 106 colon patients. During the first five years of follow-up, 34.6% patients succumbed to the disease as a result of disease recurrence. The five-year overall survival rate was 65.4%. Univariate analysis revealed that T stage and pathological type were risk factors associated with the five-year survival of colon cancer patients. In patients with stage T_3_N_1_M_0_ carcinoma the five-year survival rate was 69.6%, while in patients at stage T_4_N_1_M_0_ it was 33.3 % (P<0.001). The five-year survival rate of patients with papillary and tubular adenocarcinoma was 68.3% and it was 54.5% in patients with mucinous adenocarcinoma and signet-ring cell carcinoma. Additional clinicopathological characteristics, including age, gender, tumor location and mTOR and pmTOR expression, were not associated with survival outcomes. However, a high expression of pmTOR along tumor borders was considered to contribute to a higher risk of mortality in patients. The five-year survival rates of patients with pmTOR expression in the tumor invasion front and surrounding the tumor center were 60.6% and 78.9%, respectively (Table II).

**Figure 3:**
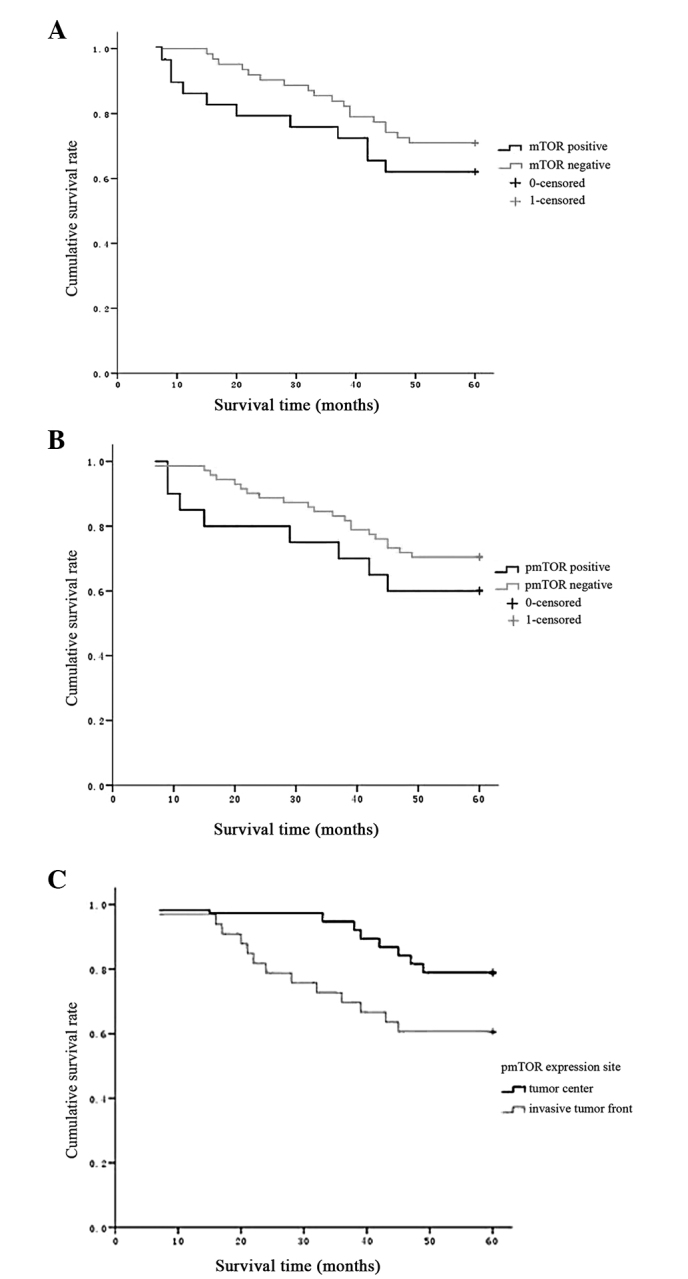
Association between overall survival and mTOR and pmTOR expression in stage IIIB colon cancer patients. mTOR, mammalian target of rapamycin; p, phosphorylated.

In multivariate analysis (Table III), TNM and pathological type were found to be independent prognostic factors for colorectal cancer patient survival. Patients with high pathological stage exhibited a higher risk of mortality than those with low stage [partial regression coefficient (B), 1.611; relative risk (RR), 5.017; 95% confidence interval (CI), 1.95–12.91; P<0.001]. Patients with papillary adenocarcinoma and tubular adenocarcinoma tended to exhibit a high survival rate (B, 0.871; RR, 2.391; 95% CI, 1.02–5.56; P=0.044). The age, gender, tumor location, and mTOR and pmTOR expression were not associated with survival outcomes (Fig. 3).

## Discussion

mTOR is a serine/threonine kinase that mediates multiple intracellular signaling pathways, which is important in the regulation of cell growth, proliferation and survival in response to energy and nutrient levels (13,14). mTOR signals via two effector branches, the TOR complex (TORC) 1 and 2 pathways, functioning by regulating protein synthesis, which affects cell growth and proliferation (15). In mammalian cells, mTORC1 controls mitochondrial transcriptional regulator peroxisome proliferator-activated receptor gamma coactivator 1-α, which is important in mitochondrial biogenesis (16). Rapamycin may inhibit mTORC1 and mTORC2 pathways, lipogenesis and glycolysis, resulting in the inhibition of proliferation and induction of apoptosis in colon cancers (17,18). Inhibition of mTORC1 activity by rapamycin has been shown to decrease mitochondrial gene expression (19). mTORC2 is a key regulator of the actin cytoskeleton and regulates the AGC kinase subfamily, which includes Akt (20). Increased Akt activity has been associated with various diseases, including cancer and diabetes. Furthermore, the Akt/mTOR signaling pathway is frequently altered in certain types of cancer, including gastric cancer, prostate cancer, cervical carcinoma, renal cell carcinoma, lung carcinoma and pancreatic ductal adenocarcinoma (21–25). Activated mTOR (pmTOR) has been shown to be associated with tumors in a number of cancer tissues. In addition, previous studies have found that the levels of mTOR and pmTOR expression were elevated in extrahepatic cholangiocarcinoma and high-grade cervical squamous cell carcinoma, respectively, when compared with normal cervical epithelium (26–28).

In this study, we detected that the positive rate of mTOR and pmTOR expression was significantly higher in 75.5% (80/106) and 76.4% (81/106) of the 106 colon cancer specimens, compared with the adjacent normal tissues. Furthermore, univariate and multivariate analysis identified no correlation between mTOR and pmTOR expression and survival rate or prognosis of patients with locally advanced colon cancer, which was consistent with the results of Tampellini *et al* (29). We hypothesize that hyperactivation of the mTOR pathway may only affect proliferation and angiogenesis during the advanced stages of colon cancer; however, it may not affect patient survival, as a result of present comprehensive intervention treatment methods.

Dobashi *et al* (30) reported that pmTOR expression in lung adenocarcinoma specimens was found to correlate with the grade of histological differentiation, whereas the pmTOR expression in squamous cell carcinoma specimens was found to correlate with lymph node metastasis. Yu *et al* (31) demonstrated that mTOR overexpression was associated with high and moderate differentiation, T1/T2 tumors and stage I/II disease, whereas pmTOR overexpression was found to correlate with lymph node metastasis and advanced-stage disease, and may present an independent predictor of gastric cancer survival. mTOR and pmTOR are useful biomarkers for predicting outcome. In this study, high pmTOR expression was detected in the forefront of tumor-infiltrating cells, resulting in an increased mortality rate of colon cancer patients. However, no significant correlation between mTOR and pmTOR overexpression in stage IIIB colon cancer and survival time and TNM staging were identified.

In conclusion, the present study indicated that mTOR and pmTOR were significantly expressed in patients with stage IIIB colon cancer, and a high expression of pmTOR was detected in the forefront of tumor-infiltrating cells, increasing the mortality rate of colon cancer patients. The results of the present study indicated that mTOR and pmTOR may be important in colorectal carcinoma and may present promising novel molecular targets for designing novel therapeutic strategies to control colorectalcarcinoma.

## Figures and Tables

**Table I:** Correlational analysis of mTOR and pmTOR expression and the clinicopathological characteristics of 106 patients with colon carcinoma stage IIIB.

	Gender	Age	Primary tumor site	Tumor grade	Pathological type	mTOR expression	TNM stage	pmTOR expression
Gender								
r	1	0.115	0.035	0.112	−0.103	0.010	−0.059	0.109
P-value	-	0.271	0.756	0.294	0.276	0.887	0.563	0.291
Age								
r	0.118	1	0.029	0.034	0.018	−0.045	−0.020	−0.125
P-value	0.268	-	0.983	0.778	0.872	0.671	0.850	0.242
Primary tumor site								
r	0.036	0.005	1	−0.012	0.071	−0.088	−0.014	−0.072
P-value	0.778	0.988	-	0.910	0.530	0.408	0.879	0.503
Tumor grade								
r	0.109	0.034	−0.012	1	0.276	−0.026	−0.025	0.085
P-value	0.279	0.751	0.912	-	0.079	0.802	0.832	0.419
Pathological type								
r	−0.103	0.019	0.070	0.273	1	0.078	−0.116	0.079
P-value	0.277	0.889	0.503	0.009	-	0.463	0.277	0.398
mTOR expression								
r	0.020	−0.045	−0.088	−0.036	0.077	1	−0.035	0.789
P-value	0.931	0.667	0.389	0.829	0.459	-	0.731	<0.001
TNM stage								
r	−0.066	−0.019	−0.017	−0.026	−0.125	−0.042	1	−0.046
P-value	0.574	0.850	0.87	0.802	0.277	0.738	-	0.665
pmTOR expression								
r	0.120	−0.135	−0.068	0.087	0.088	0.775	−0.057	1
P-value	0.286	0.251	0.511	0.403	0.340	<0.001	0.665	-

-, no data; r, correlation coefficient; mTOR, mammalian target of rapamycin; pmTOR, phosphorylated mTOR.

**Table II:** Univariate analysis of overall survival in 106 patients with stage IIIB colon carcinoma.

	Cases (n=106)	Five-year survival rate (%)	P-value
Gender		65.4	0.635
Male	67	62.0	
Female	39	70.9	
Age, years		65.5	0.072
≥60	57	75.2	
<60	49	57.2	
Primary tumor site		65.8	0.219
Left colon	64	71.2	
Right colon	42	56.5	
Pathological type		65.3	0.036
Tubular and papillary adenocarcinoma	83	68.7	
Mucinous and signet ring cell carcinoma	23	54.8	
Tumor grade		65.5	0.179
G_1_	6	80.1	
G_2_	80	68.7	
G_3_	20	50.3	
TNM stage		65.4	0.010
T_3_N_1_M_0_	93	69.6	
T_4_N_1_M_0_	13	33.3	
mTOR expression		65.4	0.294
Positive	90	62.1	
Negative	16	70.1	
pmTOR expression		65.4	0.295
Positive	99	58.2	
Negative	18	70.4	

mTOR, mammalian target of rapamycin; p, phosphorylated.

**Table III:** Multivariate analysis of overall survival in 104 patients with stage IIIB colon carcinoma using the Cox regression model.

Variable	B	SE	Wald χ^2^	Df	Unilateral	RR	95% CI
Age	1.051	0.421	6.197	1	0.013	0.351	0.15–0.80
TNM stage	1.611	0.480	11.201	1	0.001	5.017	1.95–12.91
Pathological type	0.871	0.435	4.053	1	0.044	2.391	1.02–5.56

B, partial regression coefficient; SE, standard error; Df, degrees of freedom; RR, Relative risk; CI, confidence interval.
